# A simple technique to help avoid varus malreduction of reverse oblique proximal femoral fractures

**DOI:** 10.1308/003588413X13511609957056b

**Published:** 2013-01

**Authors:** DJ Westacott, S Bhattacharaya

**Affiliations:** Heart of England NHS Foundation Trust, UK

## Background

Clinical guidelines from the National Institute for Health and Clinical Excellence recommend the use of an intramedullary nail for the treatment of subtrochanteric fractures.^1^ Reverse oblique fractures are associated with varus malunion,^2^ which defunctions the abductors by placing them at a mechanical disadvantage and increases the lever arm across the fracture. Most modern nails have been designed with a lateral bend to accommodate a trochanteric entry point. This simple technique helps to avoid varus malreduction without the need for excessive traction or open reduction.

## Technique

**Figure 1:**
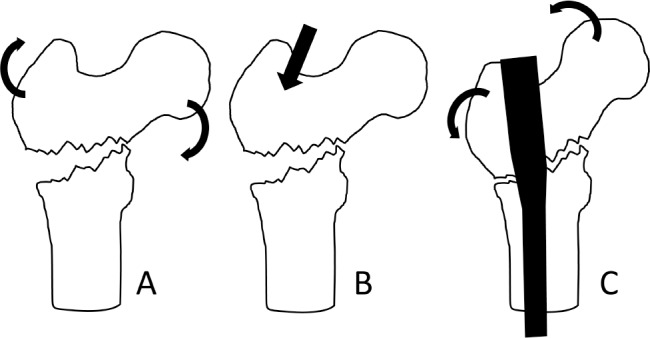
Reverse oblique fractures are prone to varus angulation (A). A medialised entry point (B) corrects the varus when the nail engages the distal fragment (C).

**Figure 2:**
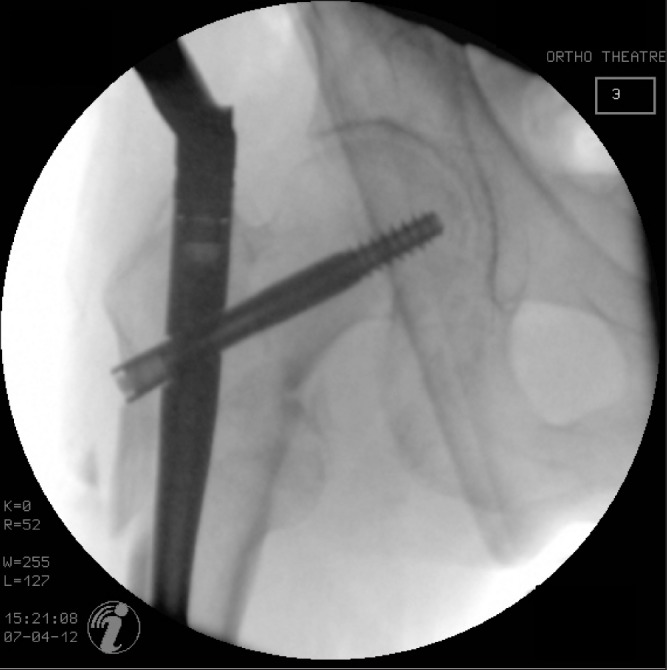
A more medial entry point has corrected the neck shaft angle but some lateral translation of the proximal fragment has occurred.

A routine preparation and approach to the greater trochanter is performed. The entry point is positioned medial to the tip of the trochanter. The cortex is breached and the guidewire passed in a direction aiming slightly from medial to lateral (Fig 1). This means that when the nail is inserted, it will engage with the lateral cortex of the distal fragment and rotate the proximal fragment into valgus, closing the fracture gap and restoring anatomy (Fig 2).

## Discussion

Anatomical closed reduction of a reverse oblique fracture is difficult due to the pull of the abductors. Abducting the leg is often attempted to restore the normal neck shaft angle but this makes access for the procedure more difficult. We describe a simple alteration of surgical technique that can prove very useful in preventing varus malunion, improving functional outcome and speed of union. Readers should be aware that medialising the entry point without angulating the direction of entry leaves the potential for lateral translation, rather than rotation, of the proximal fragment (Fig 2).
